# Hybrid Framework for Cartilage Damage Detection from Vibroacoustic Signals Using Ensemble Empirical Mode Decomposition and CNNs

**DOI:** 10.3390/s25216638

**Published:** 2025-10-29

**Authors:** Anna Machrowska, Robert Karpiński, Marcin Maciejewski, Józef Jonak, Przemysław Krakowski, Arkadiusz Syta

**Affiliations:** 1 Department of Machine Design and Mechatronics, Faculty of Mechanical Engineering, Lublin University of Technology, Nadbystrzycka 36, 20-618 Lublin, Poland; 2 Institute of Medical Sciences, Faculty of Medicine, The John Paul II Catholic University of Lublin, ul. Konstantynów 1H, 20-708 Lublin, Poland; 3 Department of Electronics and Information Technology, Faculty of Electrical Engineering and Computer Science, Lublin University of Technology, Nadbystrzycka 36, 20-618 Lublin, Poland; 4 Department of Trauma Surgery and Emergency Medicine, Medical University of Lublin, Staszica 11, 20-081 Lublin, Poland; 5 Orthopaedic and Sports Traumatology Department, Carolina Medical Center, Pory 78, 02-757 Warsaw, Poland; 6 Department of Technical Computer Science, Faculty of Mathematics and Technical Computer Science, Lublin University of Technology, Nadbystrzycka 38, 20-618 Lublin, Poland; a.syta@pollub.pl

**Keywords:** vibroarthrography, osteoarthritis, kinetic chain, detrended fluctuation analysis

## Abstract

This study proposes a hybrid analytical framework for detecting chondromalacia using vibroacoustic (VAG) signals from patients with knee osteoarthritis (OA) and healthy controls (HCs). The methodology combines nonlinear signal decomposition, feature extraction, and deep learning classification. Raw VAG signals, recorded with a custom multi-sensor system during open (OKC) and closed (CKC) kinetic chain knee flexion–extension, underwent preprocessing (denoising, segmentation, normalization). Ensemble Empirical Mode Decomposition (EEMD) was used to isolate Intrinsic Mode Functions (IMFs), and Detrended Fluctuation Analysis (DFA) computed local (α_1_) and global (α_2_) scaling exponents as well as breakpoint location. Frequency–energy features of IMFs were statistically assessed and selected via Neighborhood Component Analysis (NCA) for support vector machine (SVM) classification. Additionally, reconstructed α_1_/α_2_-based signals and raw signals were converted into continuous wavelet transform (CWT) scalograms, classified with convolutional neural networks (CNNs) at two resolutions. The SVM approach achieved the best performance in CKC conditions (accuracy 0.87, AUC 0.91). CNN classification on CWT scalograms also demonstrated robust OA/HC discrimination with acceptable computational times at higher resolutions. Results suggest that combining multiscale decomposition, nonlinear fluctuation analysis, and deep learning enables accurate, non-invasive detection of cartilage degeneration, with potential for early knee pathology diagnosis.

## 1. Introduction

As society ages, the number of cases of musculoskeletal disorders increases, which poses a significant challenge for healthcare systems. Knee osteoarthritis is one of the most common musculoskeletal conditions, leading to chronic pain, limited mobility, and a reduced quality of life for patients [1,2,3,4]. Osteoarthritis (OA) is a chronic, progressive disease characterized by degeneration of the articular cartilage, changes in the subchondral bone layer, osteophyte formation, and inflammation of the synovial membrane [5,6,7,8]. It most commonly affects the knee, hip, and hand joints, leading to pain, stiffness, and limited motor function. The pathological process develops over many years and may initially be asymptomatic, making early detection difficult. In advanced stages, the disease significantly impairs quality of life and often requires surgical intervention, including joint replacement. Therefore, methods for identifying functional changes in the early stages of the disease have become particularly important. Early detection of these changes is crucial for effective treatment before irreversible cartilage degeneration occurs [9,10]. Traditional diagnostic methods, such as magnetic resonance imaging (MRI) and arthroscopy, while effective, are associated with high costs, limited availability, or an invasive nature. As a result, there is growing interest in alternative, non-invasive methods for assessing the functional status of joints, based on the analysis of biomechanical signals generated during joint movement [11,12,13].

Vibroarthrography (VAG) is a non-invasive diagnostic method based on the analysis of mechanical vibrations generated by moving synovial joints, which are recorded using acoustic or vibration sensors placed on the skin near the joint [14,15]. These signals reflect microvibrations resulting from the contact of articular surfaces, muscle tension, and biomechanical interactions during flexion and extension of the limb. VAG is used for functional assessment of the knee joint and shows potential for detecting early-stage cartilage pathology. Compared to imaging methods, VAG offers high availability, low cost, and the ability to conduct dynamic assessments (which is not possible with imaging diagnostics), making it an attractive tool to support orthopedic diagnostics [16]. However, the complex, nonlinear, and non-stationary nature of VAG signals presents a challenge, requiring advanced analytical methods to identify diagnostically relevant features.

Previous research on vibroarthrographic (VAG) signals has largely utilized traditional analysis techniques, for instance, the Fast Fourier Transform (FFT), envelope analysis, and band-pass filtration. Despite their potential for an initial frequency characteristic analysis of the signal, these techniques demonstrate principal shortcomings when applied to signals that show time-variable structures, for example, biological signals. [15,16,17]. VAG signals are inherently non-stationary, exhibit multiscale fluctuations, and are strongly influenced by biomechanical and neuromuscular factors. As a result, linear methods may overlook subtle dynamic and correlational changes that could be crucial for identifying pathological conditions [18]. In recent years, there has been growing interest in techniques that enable the analysis of local signal properties in both time and frequency domains, while also accounting for the nonlinear nature of the data [19,20].

In response to the limitations of classical methods, the analysis of biological signals, including VAG signals, has increasingly incorporated advanced nonlinear and multiscale signal processing techniques. Among these, Ensemble Empirical Mode Decomposition (EEMD) stands out as an extension of the classical EMD approach. EEMD enables the adaptive decomposition of a signal into Intrinsic Mode Functions (IMFs) with distinct frequency characteristics while preserving its local temporal structure [21,22]. This analysis is often complemented by Detrended Fluctuation Analysis (DFA), which allows for the assessment of temporal correlations within the signal across both short-term (α_1_) and long-term (α_2_) time scales.

Furthermore, the use of deep learning techniques, particularly Convolutional Neural Networks (CNNs), has become increasingly prevalent. These methods enable the classification of time–frequency representations (e.g., those obtained via wavelet transform) without the need for manual feature extraction [23]. The integration of these modern approaches opens new avenues for the precise analysis of VAG signals and the development of objective diagnostic tools.

Despite advancements regarding biomechanical signal analysis, approaches remain lacking that fully account for the non-stationary, nonlinear nature and multiscale time–frequency structure of vibroarthrographic (VAG) signals. Many current investigations use separate procedures that involve standard frequency-domain processes or specific feature extraction formulas without thinking about the possible interaction between updated separation strategies, nonlinear correlation examination, and mechanical sorting using deep neural networks. Furthermore, studies do not, in fact, examine the α_1_ and α_2_ features that Detrended Fluctuation Analysis (DFA) derives in the context of specific Intrinsic Mode Functions (IMFs) that Ensemble Empirical Mode Decomposition (EEMD) obtains, nor do studies compare the classification performance that Convolutional Neural Networks or CNNs achieve. This research addresses the aforementioned gap by coherently proposing a dual-path approach that extracts nonlinear features and analyzes time–frequency images. We aim to distinguish more accurately between pathological and physiological joint conditions. We also aim to identify potential diagnostic markers that can be implemented in medical decision support systems.

The purpose of this research was to design and validate a new hybrid analytical system for detecting degenerative cartilage changes in the knee joint non-invasively. This proposed hybrid analytical system brings together current advances in nonlinear signal decomposition, multiscale fluctuation analyses, and image-based classification with machine learning algorithms. The research investigates a hybrid method:

(i) The extraction of diagnostically relevant features in the frequency domain from vibroacoustic signals (VAGs) was performed using the Ensemble Empirical Mode Decomposition (EEMD) method and dual-band Detrended Fluctuation Analysis (DFA). The extracted features were subsequently used for classification, the results of which are presented in the following sections of this study.
(ii) The classification of scalograms from Continuous Wavelet Transform (CWT) analyses on both the raw signals and the signals reconstructed based on qualitatively defined alternating α_1_/α_2_ correlation patterns was conducted via Convolutional Neural Networks (CNNs).

This bidirectional methodology allows for the simultaneous exploration of complementary feature spaces, both quantitative descriptors and time–frequency patterns provided in an image. This improves classification metrics, including precision, robustness to biomechanical variability, and efficiency, thereby enabling the basis for future real-time application within intelligent clinical decision support systems for early detection of knee joint pathologies.

## 2. Materials and Methods

### 2.1. Study Participants

The study was conducted on a group of 97 adult volunteers (41 males and 56 females) with a mean age of 41.67 years. Recruitment and participant assessment were carried out at two centers: the Clinic of Trauma Surgery and Emergency Medicine at the Medical University of Lublin and the laboratories of the Lublin University of Technology.

Participants were divided into two groups:

- OA group (osteoarthritis)—consisting of patients diagnosed with knee osteoarthritis who were qualified for surgical treatment (arthroscopy or total knee arthroplasty). Qualification was based on medical history, physical examination, and radiological assessment conducted by a specialist in orthopedics and trauma surgery of the musculoskeletal system.
- HC group (healthy control)—comprising healthy volunteers with no prior history of pain, injuries, or diagnosed pathological changes in the knee joint.

Inclusion criteria for the OA group were (i) clinical and radiological diagnosis of knee osteoarthritis, (ii) qualification for arthroscopy or total knee arthroplasty based on orthopedic evaluation, (iii) intraoperative confirmation of focal cartilage lesions, and (iv) provision of informed consent to participate in the study. The control group included volunteers with no clinical symptoms of joint diseases or history of prior injuries.

**Exclusion criteria for both groups included** (i) previous surgical procedures involving the knee joint, (ii) positive orthopedic examination findings in the control group, (iii) confirmed or suspected ligament injuries, (iv) visible axial limb deformities or gait abnormalities, and (v) neuromuscular or systemic diseases affecting joint function.

This study was designed as a pilot investigation, aimed at verifying the feasibility of vibroacoustic signal analysis in differentiating joint conditions under controlled clinical circumstances. The OA group consisted of patients qualified for arthroscopy or total knee arthroplasty, which implies that most participants represented advanced stages of degeneration (Kellgren–Lawrence grades 3–4). Nevertheless, the inclusion process was preceded by a comprehensive physical examination performed by an orthopedic surgeon, who also evaluated patient eligibility both for surgery and for participation in the project.

Such dual assessment ensured the accuracy of clinical diagnosis and minimized the influence of potential confounding musculoskeletal conditions. While the present dataset primarily represents moderate to advanced OA, the methodological framework was established with the goal of future expansion toward early OA stages (grades 1–2) and multi-level classification of cartilage damage, as also outlined in Section 6.

In the OA group, all vibroarthrographic (VAG) signal measurements and recordings were performed one day prior to the scheduled surgical procedure, allowing for the minimization of the time interval between diagnostic assessment and treatment. The study received a positive ethical approval from the Bioethics Committee of the Medical University of Lublin, approval number KE-0254/261/2019.

### 2.2. Measurement System and Acoustic Signal Recording

For the recording of vibroacoustic signals (VAGs) generated by the knee joint during movement, a custom measurement system based on the Arduino Mega2560 R3 microcontroller was used. The acoustic signal was collected using three piezoelectric contact microphones of the CM01B type, attached directly to the skin with double-sided tape in the areas of the patella as well as the medial and lateral femoral condyles. To measure the knee flexion angle, an EMS22A50-D28-LT6 encoder (Bourns, Inc., Riverside, CA, USA) was mounted on the axis of the Breg T-Scope Knee orthosis (Breg, Inc., Carlsbad, CA, USA). The system was battery-powered, and user safety was ensured by a galvanic isolation barrier on the USB interface. The device design was lightweight, mobile, and adapted for use in both clinical and laboratory settings. A detailed description of the measurement system is provided in references [24,25].

**Figure 1:**
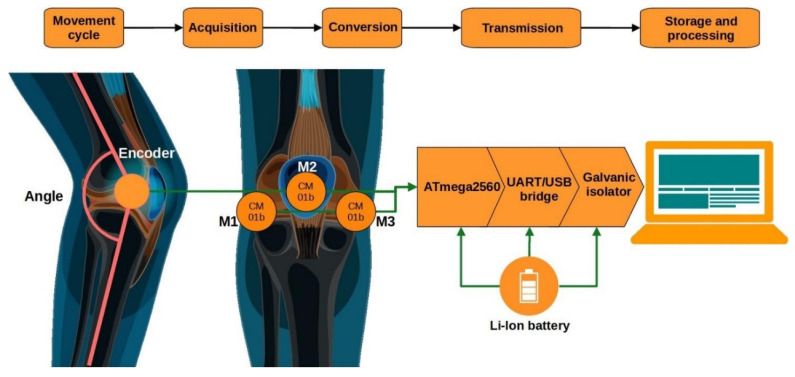
Block diagram of the system for acoustic signal acquisition.

The study was conducted in two groups: a control group (healthy individuals) and a patient group (patients previously diagnosed with knee osteoarthritis). In both cases, a medical interview and physical examination were performed prior to measurements. Signal recording in the patient group was carried out one day before the scheduled surgical procedure, whereas in the control group it was conducted under laboratory conditions. The block diagram of the measurement system for acoustic signal acquisition is presented in Figure 1.

Signals were recorded during the performance of 10 cycles of knee flexion and extension within a range of 0–90° under two movement conditions: open kinetic chain (OKC) and closed kinetic chain (CKC). In the OKC condition, participants sat freely and performed alternating knee extension and flexion. In the CKC condition, the examination was carried out during a controlled squat without additional load. The collected data allowed for the analysis of signal changes in relation to movement biomechanics and the condition of the articular surfaces. The acquired signals were subsequently subjected to further processing.

The influence of sensor placement on signal quality and diagnostic accuracy was analyzed and discussed in the authors’ previous studies on vibroacoustic assessment of the knee joint [26,27]. In these investigations, the optimal sensor configuration for both the femoro-tibial and patellofemoral joints was experimentally validated. It was shown that sensors positioned directly above the medial and lateral femoral condyles and on the patellar apex provide the highest signal-to-noise ratio (SNR) and the greatest repeatability between trials, while maintaining patient comfort during dynamic flexion–extension cycles.

Minor displacements of the contact microphones (up to 5 mm) did not significantly affect diagnostic indices such as RMS amplitude or dominant frequency, which confirms the robustness of the proposed setup. However, the signal quality rapidly decreased when sensors were shifted toward areas with thicker soft-tissue coverage, mainly due to attenuation and phase delay. Consequently, in the current study, the same standardized configuration was used for all participants, as detailed in earlier works [26,27].

Additionally, all sensors were fixed using double-sided medical adhesive tape to minimize relative motion artifacts. The consistent placement relative to bony landmarks ensured comparability of VAG recordings across subjects and measurement sessions. The methodology thus maintains high reproducibility and accuracy in the assessment of vibroacoustic features related to articular cartilage status.

### 2.3. Preliminary Signal Processing

The recorded vibroacoustic (VAG) signals were subjected to preprocessing aimed at ensuring data comparability and removing noise unrelated to the analyzed joint activity. To standardize the length of the analyzed signals and eliminate the influence of stationary segments, each recording was trimmed to a portion corresponding to 10 movement cycles, both in the open kinetic chain (OKC) and closed kinetic chain (CKC) conditions. A custom algorithm was implemented to detect movement cycles based on changes in signal slope, enabling automatic and precise segmentation of active motion. To remove low-frequency noise, a fourth-order Butterworth digital filter with a cutoff frequency of 10 Hz was applied [28]. This approach preserved components related to the physiological activity of the joint while eliminating vibrations caused by environmental factors or hardware-related disturbances. For further analysis, the signals were normalized to the range [0, 1], which reduced the influence of individual amplitude differences resulting from factors such as body mass, applied pressure, or recording conditions. Normalization also improved numerical stability during subsequent processing stages, such as time–frequency transformations and statistical analysis. The signals obtained at this stage were considered raw waveforms.

## 3. Hybrid Analysis of Vibroacoustic Signals of the Knee Joint

This chapter presents a detailed procedure for the acquisition, processing, and analysis of vibroacoustic signals (VAGs) recorded from the knee joint. It describes the participant selection criteria, the measurement system used, and the experimental conditions. Subsequently, the successive stages of signal processing are outlined, including filtering, segmentation, and decomposition using the Ensemble Empirical Mode Decomposition (EEMD) method. The chapter further discusses the analysis of the nonlinear properties of the signal using Detrended Fluctuation Analysis (DFA), as well as strategies for feature reduction and selection.

### 3.1. Frequency Filtering Using the Ensemble Empirical Mode Decomposition (EEMD) Method

The next stage, following the initial signal preprocessing, was frequency filtering using the Ensemble Empirical Mode Decomposition (EEMD) method. EEMD is an extension of the classical Empirical Mode Decomposition (EMD), designed to improve the stability of the decomposition of nonlinear and non-stationary signals. Traditional EMD can be sensitive to noise and prone to the so-called “mode mixing” phenomenon, where different frequency components overlap within a single Intrinsic Mode Function (IMF) [29,30]. EEMD addresses this issue by adding low-amplitude white noise to the signal and performing multiple decompositions, each time using a different noise realization. The final result is obtained by averaging the individual decompositions, which reduces the influence of noise while preserving the true structure of the signal.

In the analysis, the ratio of the standard deviation of the added noise to the standard deviation of the input signal was set at 0.01, meaning that the added noise accounted for only 1% of the variability of the original signal. This parameter choice is consistent with the recommendations of Wu and Huang [31,32], who demonstrated that a low-level noise of approximately 0.1–1% of the signal amplitude is sufficient to mitigate mode mixing while preventing distortion of the intrinsic dynamics of the signal. Lower noise levels do not effectively separate oscillatory modes, whereas higher levels introduce artificial fluctuation patterns.

The number of ensemble realizations was set to 100, which falls within the range suggested in EEMD performance studies (typically 50–200 realizations) as a balance between decomposition stability and computational efficiency [31,32]. After performing 100 independent decompositions with different noise realizations, the resulting IMFs were averaged to provide a stable representation of the signal components with minimized influence of stochastic variability. As a result, the obtained IMFs retained their physical interpretability while minimizing the impact of noise-induced artifacts.

### 3.2. Exploratory Analysis of Components

To ensure reliable classification of the knee joint health status based on vibroacoustic signals, an exploratory analysis of the frequency components obtained by the EEMD method was conducted. Due to the nonlinear and non-stationary nature of VAG signals, the Intrinsic Mode Functions (IMFs) exhibit diverse time–frequency structures that may significantly differ between healthy individuals and patients with osteoarthritis. Analysis of parameters such as centroid frequency, instantaneous frequency, and energy of each component enabled the identification of those components potentially containing diagnostically relevant information. This approach allowed for the preliminary localization of frequency bands most critical for differentiating pathological states, as well as the assessment of signal stability and intensity within selected ranges. Based on the extracted parameters, a classification process was also performed, aiming at automatic discrimination between healthy and pathological conditions. The application of these features in classification models allowed for the evaluation of their diagnostic utility and confirmed their significance in differentiating between the OA and HC groups. The obtained results also provided a necessary foundation for further correlation analyses using the DFA method, ensuring that the analyzed components possessed both meaningful physiological relevance and adequate signal quality. Thus, the exploratory analysis played a key role in optimizing the subsequent processing stages and interpreting the classification outcomes.

The signals obtained through EEMD from individuals diagnosed with osteoarthritis and healthy subjects exhibited slight frequency differences, which is entirely natural and consistent with the adaptive nature of the EEMD method. This also reflects the differing frequency structures of signals from healthy and diseased individuals, attributable to variations in movement patterns, muscle fluctuations, vibrations, and tension. To obtain reliable results for characterizing the collected signals and constructing healthy–diseased classification profiles, an exploratory analysis of the frequency components was conducted. For the assessment of signal properties and their time–frequency distribution, three parameters were extracted from each IMF component: centroid frequency, mean instantaneous frequency, and energy.

The centroid frequency is the weighted average of frequencies, and its value helps determine where the dominant frequency activity is located within the frequency spectrum. It is calculated as follows:

(1) $$CFr=\frac{\sum f\cdot P\left(f\right)}{\sum P\left(f\right)},$$

where *f* denotes the frequency, and *P*(*f*) represents the power spectral density (PSD).

**Figure 2:**
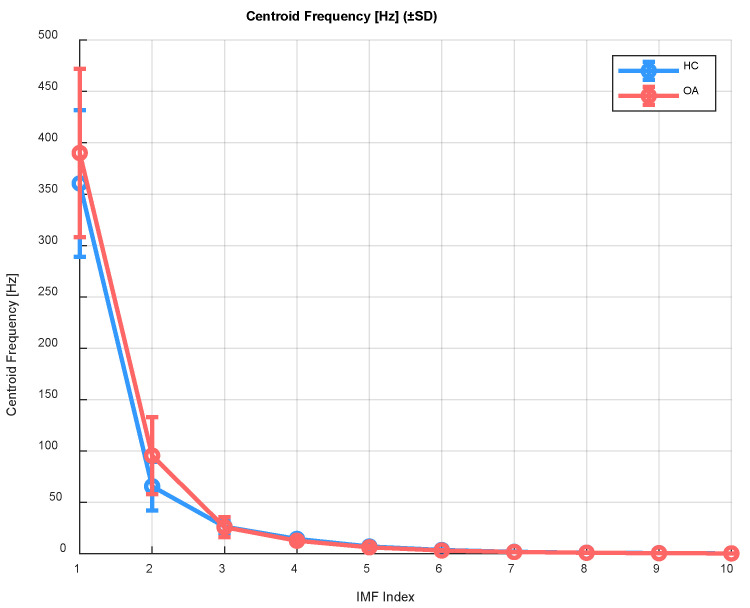
Centroid Frequency as a Function of IMF Indices for HC and OA Groups, Patellofemoral Joint, CKC.

In the context of further signal processing in the conducted study, it allows the level of frequency activity within the given IMFs to be determined. For example, Figure 2 presents the calculated centroid frequency values along with the standard deviation (±SD) as a function of the IMF indices for the OA and HC groups, specifically for the patellofemoral joint (acoustic signals).

The analysis of centroid frequency across successive IMF components of the vibroarthrographic signal revealed significant differences between the control group (HC) and patients with knee osteoarthritis (OA). The highest centroid frequency values were observed in the initial IMF components (IMF 1–3), consistent with the presence of high-frequency components in the early decomposition modes. For IMF components 1 and 2, centroid frequency values were significantly higher in OA patients compared to healthy individuals, which may indicate increased friction, roughness of joint surfaces, and cartilage degeneration. Significant differences between groups were also observed in the subsequent IMFs (corresponding to mid and lower frequencies), despite lower absolute values. This may reflect the influence of lower-frequency components associated with mechanical limb movement and neuromuscular activity. These findings justify the application of further quantitative signal analysis methods, such as Detrended Fluctuation Analysis (DFA), to assess local (α_1_) and global (α_2_) temporal correlations in the VAG signal. This approach allows for a deeper understanding of the dynamics and structure of temporal fluctuations, which may vary depending on the presence of joint pathology. The observed differences in centroid frequency suggest potential changes in the spectral characteristics of VAG signals related to joint health status. To further investigate this aspect and evaluate frequency variability over time, instantaneous frequency calculations were performed, enabling the capture of dynamic signal properties with higher temporal resolution.

**Figure 3:**
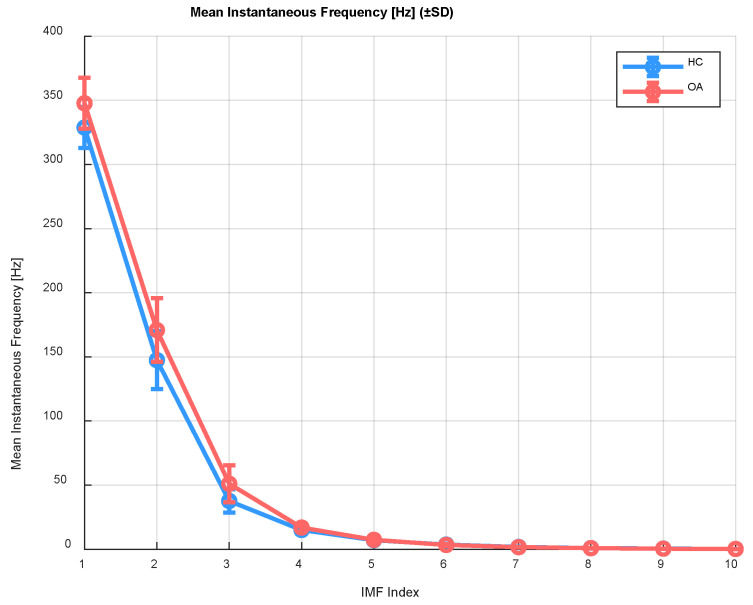
Mean Instantaneous Frequency as a Function of IMF Indices for HC and OA Groups, Patellofemoral Joint, CKC.

The mean instantaneous frequency indicates whether faster or slower frequency changes predominate in the analyzed signal over time. It was calculated from the phase of the analytic signal as follows:

(2) $$f_{inst}\left(t\right)=\frac{1}{2\pi }\cdot \frac{d}{dt}\arg\left(x_{a}\right),$$

where *arg*(*x_a_*) denotes the phase of the analytic signal (obtained by applying the Hilbert transform to the input signal *x_IMF_i_*. Differences in instantaneous frequency may indicate changes in movement dynamics between healthy individuals and patients. For example, individuals with osteoarthritis may exhibit lower frequency variability (smaller amplitudes of instantaneous frequency fluctuations) compared to healthy subjects, which could suggest reduced mobility or alterations in movement patterns. Similarly to the centroid frequency, a comparative analysis of mean instantaneous frequency values was performed for the OA and HC groups, exemplified by the patellofemoral joint in a closed kinematic chain (Figure 3).

The analysis of the mean instantaneous frequency revealed significant differences between the control group (HC) and patients with knee osteoarthritis (OA). Elevated instantaneous frequency values in the initial IMF components (IMF 1–3) observed in OA patients may indicate the presence of irregularities in joint surfaces and increased friction, characteristic of cartilage degeneration. Differences in mean frequencies within IMF ranges 4–6 (approximately 150–300 Hz) suggest that OA affects not only joint structure but also the execution and control of movement. Despite low absolute values in the higher IMFs (IMF 7–10, with frequencies below 150 Hz), the presence of significant differences may indicate alterations in the slower signal components, potentially related to neuromuscular compensation or altered movement dynamics.

The energy of an IMF component is defined as the sum of the squares of the input signal:

(3) $$Energy=\sum {\left(x_{IMF\_i}\right)}^{2}.$$

**Figure 4:**
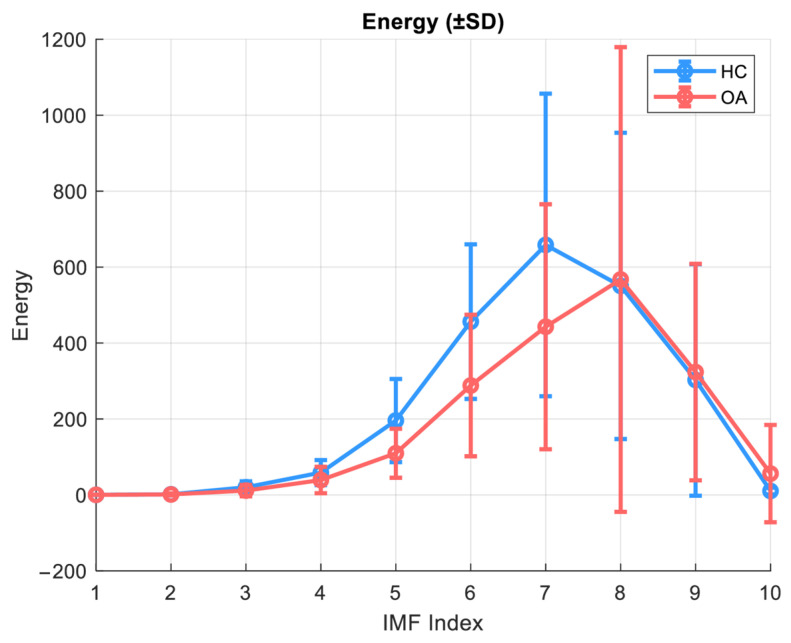
Energy as a Function of IMF Indices for HC and OA Groups, Patellofemoral Joint, CKC.

In the context of the present study, energy serves as a measure of the intensity of a given signal component, which can be crucial for building classification models and signal reconstruction in the application of Detrended Fluctuation Analysis (DFA) with separation into local and global components. Figure 4 illustrates the energy as a function of IMF indices for the OA and HC groups in the closed kinematic chain for the patellofemoral joint.

The energy analysis of individual IMF components was conducted assuming the use of 10 components. This analysis revealed significant differences between the healthy control group (HC) and patients with knee osteoarthritis (OA). In healthy subjects, the main energetic contribution of the VAG signal was concentrated in IMF 7–8, with higher maximum energy values. In contrast, the OA group exhibited not only a general reduction in energy but also a shift in the energy peak toward higher IMF indices (IMF 8), which may indicate alterations in movement biomechanics and a decreased capacity of the joint to smoothly transmit mechanical forces. In the lower, high-frequency components (IMF 1–4), the signal energy was significantly reduced in OA patients, possibly reflecting a diminished contribution of fast components associated with microvibrations and cartilage elasticity. Additionally, although the absolute values were low, significant differences in IMF 10 were observed, potentially suggesting changes in motor control or compensatory muscle activity.

The extracted parameters, such as centroid frequency, instantaneous frequency, and energy of individual IMFs, in addition to serving as feature functions used in joint condition classification, also played an auxiliary role in characterizing the signal structure and comparing the OA and HC groups. This analysis enabled:

- Preliminary identification of which components and frequency sub-bands contain the most diagnostically useful information for subsequent DFA;
- Assessment of the energy distribution across low and high frequencies and evaluation of centroid frequencies in the context of signal dynamics depending on the group;
- Estimation of the significance of individual components regarding their impact on the reliability and interpretability of DFA results;
- Determination of frequency and energy “carrier bands” related to phenomena differentiating OA from HC.

**Figure 5:**
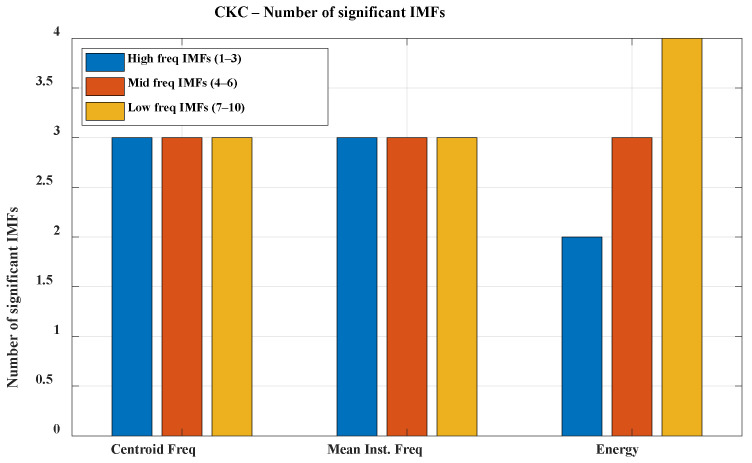
Significant IMFs across frequency bands, CKC.

**Figure 6:**
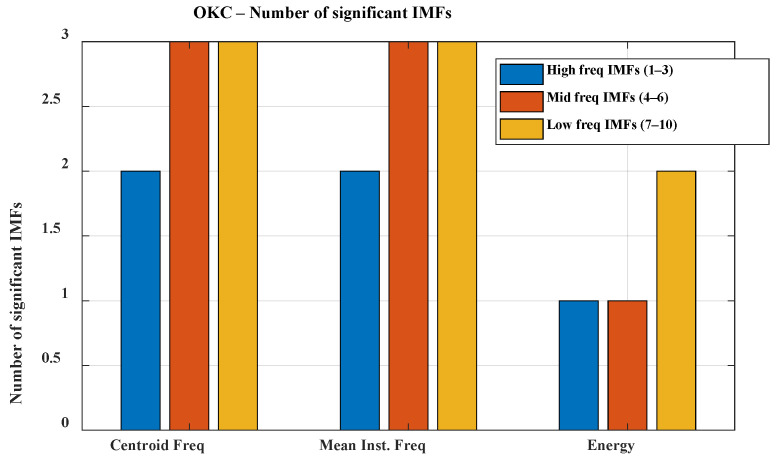
Significant IMFs across frequency bands, OKC.

The analysis was conducted separately for each experimental configuration: K1, K2, and K3 under both CKC and OKC conditions. Due to the volume of data, the direction of further analysis, and the need to simplify the interpretation of results, the individual Intrinsic Mode Functions (IMFs) were grouped into three frequency bands: high (IMF 1–3; f > 300 Hz), mid (IMF 4–6; 100 Hz < f < 300 Hz), and low (IMF 7–10; f < 100 Hz). This division allows for a clearer presentation of significant signal features and facilitates subsequent analysis; therefore, the results are presented grouped within these three bands (see Figure 5 and Figure 6).

The presented graphs illustrate the number of IMF components significant for classifying healthy (HC) and osteoarthritis (OA) groups across different frequency bands during movements performed in closed kinetic chain (CKC) and open kinetic chain (OKC) conditions. In the CKC condition, all analyzed IMFs showed significant differences between groups based on both centroid frequency and mean instantaneous frequency within the designated frequency bands. In contrast, under OKC conditions, one of the high-frequency IMF components did not exhibit significant differences, which may be attributed to biomechanical distinctions between the types of kinetic chains. High-frequency components in VAG signals are typically associated with abrupt micro-jolts, vibrations resulting from irregularities in articular surfaces, and friction, which may be less pronounced during movements executed in an open kinetic chain [33,34]. Movement performed in the open kinetic chain (OKC) is more controlled, with reduced contact and less compressive force between the articular surfaces. Consequently, the generation of sharp, high-frequency components by the joint may be limited. In contrast, movement in the closed kinetic chain (CKC) is associated with joint compression, increased load, and greater forces acting on the articular surfaces during contact. When these surfaces are irregular due to degenerative changes or chondromalacia, characteristic microvibrations occur in the higher frequency range. The high significance of high-frequency components may indicate cartilage damage, joint instability, and the presence of osteophytes. Furthermore, early degenerative changes might be signaled by an increased presence of high-frequency components exclusively in the CKC condition.

A clear relationship between frequency band range and energy is also evident. This phenomenon is natural in VAG signals and stems from the biomechanics of the knee joint. Slow movements of the articular surfaces during motion generate vibrations of lower frequency and higher amplitude. The much lower significance of mid- and high-frequency components can be explained by the attenuation of higher frequencies by the soft tissues surrounding the joint. Although high-frequency components are more specific to pathological changes, in the context of VAG signal classification for monitoring and diagnosing osteoarthritis, low-frequency components also serve an auxiliary role: assessing the general joint function, identifying movement patterns, and determining the degree of mobility limitation and biomechanical compensation. Their higher energy and resilience to disturbances caused by soft-tissue attenuation make them a stable diagnostic background that supports the interpretation of the presence or absence of pathological components in the higher frequency bands.

The presented graphs also illustrate the lower significance of all components in the energy of signals recorded during open kinetic chain movement. This phenomenon is related to reduced forces acting on the joint, limited expression of pathological changes in the signals (due to the absence of strong joint compression), a higher contribution of disturbances originating from muscle vibrations, and energy dispersion caused by a lack of stabilization in the axis of motion.

The presented results indicate that individual IMF components of the vibroarthrographic signal carry significant diagnostic information, both in terms of frequency-related and energetic parameters. The observed differences between the OA group and healthy subjects suggest that knee osteoarthritis affects not only the biomechanics of movement but also the dynamic characteristics of the VAG signal. To deepen the analysis of these differences and better understand the temporal structure of the signal, the Detrended Fluctuation Analysis (DFA) method was applied. DFA allows for the assessment of local (α_1_) and global (α_2_) temporal correlations within individual IMF components, enabling the identification of subtle changes in the temporal organization of the signal that are often not detectable using conventional frequency analysis. Of particular importance is the ability to detect alterations in motor control mechanisms and potential compensatory strategies activated in response to the progressing joint degeneration.

### 3.3. Application of Detrended Fluctuation Analysis with Dual-Scaling Exponents in Signal Characterization

Detrended Fluctuation Analysis (DFA) is a nonlinear time series analysis method originally developed to identify long-range correlations in data exhibiting features of nonlinearity, nonstationarity, and multiscale fluctuations [35,36,37]. This technique has been widely applied in the analysis of biological signals, including electroencephalographic (EEG), electrocardiographic (ECG), and electromyographic (EMG) signals, as well as biomechanical movement signals [38,39,40,41,42]. In its classical form, DFA involves dividing the cumulative time series into segments of equal length, within which local trends are removed by linear detrending. Subsequently, the variance of the detrended signal is calculated and analyzed as a function of segment length, enabling the assessment of the fluctuation characteristics of the signal across different time scales.

The basic DFA algorithm can be described in more detail as follows: For a given time series x(i) = 1, 2, …, N the cumulative sum of the differences between the individual signal values and the mean of the signal is first calculated as:

(4) $$Y\left(k\right)=\sum_{i=1}^{k}\left(x\left(i\right)-\bar{x}\right),$$

where $\bar{x}$ is the mean value of the signal. Next, *Y*(*k*) is divided into non-overlapping segments of length *n*, within which a local trend *y_n_*(*k*), typically linear, is fitted using the least squares method. The segment length *n* is often chosen from a range *n* ∊ [*n_min_, n_max_*], where *n_min_* is selected to be sufficiently large to allow reliable trend fitting, and *n_max_* is set to be smaller than the total signal length to ensure an adequate number of segments for averaging. The differences between *Y(k)* and the local trend are then used to calculate the fluctuation function:

(5) $$F\left(n\right)=\sqrt{\frac{1}{N}\sum_{k=1}^{N}{\left[Y\left(k\right)-y_{n}(k)\right]}^{2}}.$$

The relationship between *F*(*n*) and *n* typically follows a power-law function:

F(n)~n^α^, (6)

where the scaling exponent α provides information about the presence and strength of long-range correlations within the signal [43].

In the context of biological signal analysis, an extended version of DFA is increasingly employed, which includes two distinct scaling regions: local α_1_ and global α_2_ [44]. In this approach, the logarithmic relationship between *log F*(*n*) and *log n* is analyzed separately within two predefined segment length ranges, allowing for the detection of different regulatory mechanisms that dominate at short and long time scales. This extension, commonly referred to as “dual-scaling DFA” or “two-scale DFA”, enables a more precise characterization of temporal fluctuations across different timescales and has been recognized as particularly valuable in studies of motor control, postural variability, and neuromuscular dynamics [45].

Parameter α_1_, calculated over shorter time windows (e.g., 4–16 or 4–30 time points), reflects the local correlation structure and can be interpreted as a measure of short-term signal organization. In the context of motor control, higher values of α_1_ may indicate a stiffer, less adaptive form of dynamic control, whereas lower values suggest greater flexibility and variability in movement regulation [35,45,46,47]. The α_2_ parameter, computed over longer time windows (e.g., 30–300 time points), describes long-range correlations and reflects the global stability of signal dynamics. Disturbances in this domain may be indicative of pathologies associated with chronic disintegration of postural control, fatigue, or degenerative changes within the motor system [48,49,50].

The division into α_1_ and α_2_ has been successfully applied in numerous studies addressing motor system aging, Parkinson’s disease, and peripheral neuropathies, as well as gait and postural stability analysis [35,37,51]. In the context of mechanical signals originating from joints (such as vibroarthrographic signals, VAG), the application of this two-scale analysis enables the detection of subtle differences in both local disturbances of motor control (e.g., muscular compensations) and the global organization of movement, which may arise from the degradation of joint structures.

Technically, the extended DFA relies on fitting two independent linear regressions to the log–log plot of *F*(*n*) versus *n* within a selected range of segment lengths. The first range corresponds to short time scales and is used to determine the α_1_ exponent, while the second range, encompassing longer scales, yields the α_2_ exponent. The boundary between these two ranges can be established either through visual inspection (i.e., identifying a breakpoint in the scaling curve) or by applying piecewise linear regression algorithms, which enable an objective detection of changes in fluctuation dynamics.

Previous studies [21,22] have identified signal characteristics that are more appropriately described using two linear segments, capturing both local and global correlation exponents. In light of these findings, and consistent with prior results, the present study employed DFA with a two-range scaling approach (α_1_ and α_2_) applied to individual Intrinsic Mode Function (IMF) components of the VAG signal. Window lengths were selected separately for the short-term and long-term scaling ranges. Given the differing movement dynamics across the two subject groups and the variable number of data samples, an adaptive approach was adopted for selecting segment lengths *n*, based on the relative duration of the movement cycle. The minimum window size was set to 0.1 of a single cycle length, while the maximum was set to two full cycles. For each signal, 30 window lengths were defined, logarithmically spaced within the specified range, allowing for standardized interpretation of α_1_ and α_2_ across individuals, regardless of movement tempo or duration. The breakpoint separating correlation ranges was determined using a linear segmentation algorithm.

**Figure 7:**
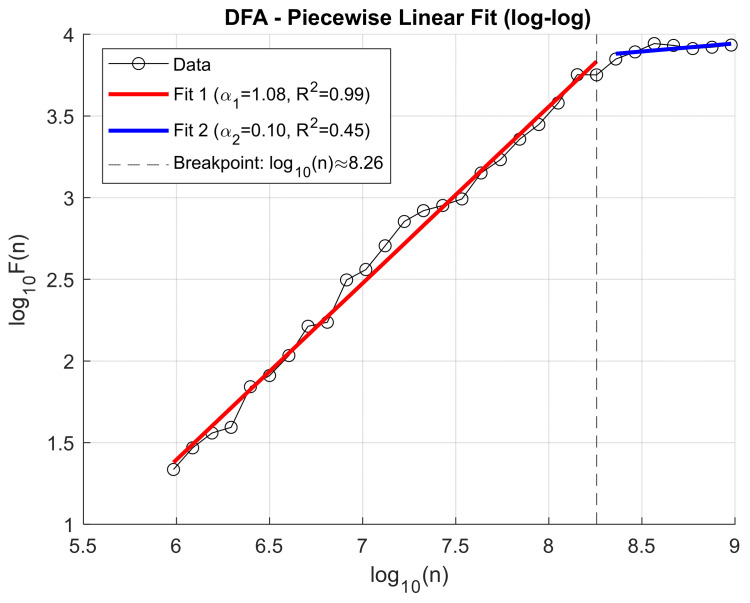
Log–log characteristic for IMF3 in an OA patient, patellofemoral joint, CKC condition; the breakpoint occurred at approximately 2.75 s (9.7% of the total signal duration).

This approach enabled a more in-depth assessment of the impact of knee osteoarthritis on the dynamic temporal structure of the signal, as well as the identification of potential diagnostic and discriminative markers. Previously, to decompose the VAG signals, the Ensemble Empirical Mode Decomposition (EEMD) method was applied, allowing for the extraction of representative Intrinsic Mode Functions (IMFs) that capture oscillations across different frequency ranges. Analysis of the fluctuation function F(n) for individual IMFs revealed that the location of the scaling transition point (breakpoint) varies both across IMF components and depending on the clinical condition of the patient. This variability may reflect differences in the dynamic characteristics of the knee joint resulting from degenerative pathologies. Illustrative figures depicting these relationships are presented below. To illustrate the fluctuation dynamics identified through Detrended Fluctuation Analysis (DFA), Figure 7 presents an exemplary log–log relationship for the third Intrinsic Mode Function (IMF3) obtained from a patient with knee osteoarthritis, recorded for the patellofemoral joint under closed kinetic chain (CKC) conditions (Figure 7). 

**Figure 8:**
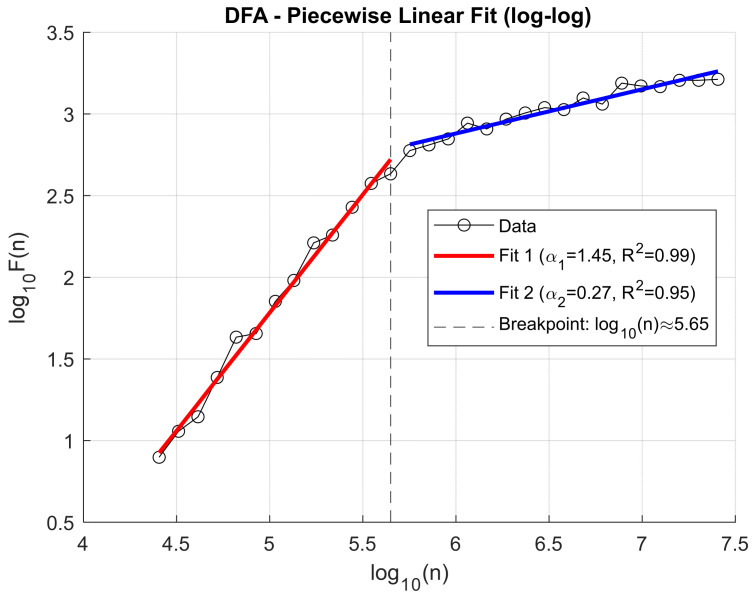
Log–log characteristic for IMF3 in a healthy control (HC), patellofemoral joint, CKC condition; the breakpoint occurred at approximately 0.2 s (3.4% of the total signal duration).

Figure 8 illustrates the log–log relationship of the fluctuation function F(n) for IMF3 obtained from a healthy control (HC) subject in the patellofemoral joint under closed kinetic chain (CKC) conditions, showing the breakpoint position that defines the transition between short- and long-range correlations.

Due to the observed difference in breakpoint location between groups, this parameter was included as an additional independent feature in subsequent classification analyses. This feature, later employed in the healthy-versus-patient classification, defines the timing of the change in slope of the DFA characteristic.

To capture changes in signal fluctuation behavior across different time scales, a piecewise linear fitting model was applied to the log–log plot of *F(n)* versus *n* (window size). This method is based on the classical Detrended Fluctuation Analysis (DFA), extended by automatic detection of the breakpoint that separates time scale ranges with distinct characteristics. The applied algorithm includes:

(a) Preprocessing of data
  - Both the window sizes *n* and the corresponding fluctuation function values *F(n)* are logarithmically transformed to obtain data in the log–log domain.
  - Zero, infinite, and non-numeric values (NaNs) that could disrupt the fitting procedure are removed.
(b) Review of all possible breakpoints
  - For each potential breakpoint *i* (position along the log(s) axis), which divides the data into two segments (with a minimum of 3 points in each segment), separate linear fits are performed:
  - Fit 1: from the beginning up to *i* (short scales).
  - Fit 2: from *i* + 1 to the end (long scales).
  - Residual sum of squares errors are computed for both fits (sum of squared differences between actual and fitted values).
(c) Selection of the optimal breakpoint
  - The point *i* yielding the lowest total residual error (combined for both segments) is selected as the breakpoint.
(d) Final fitting and quality assessment
  - The slopes of the two linear segments (corresponding to the scaling exponents α) and the coefficients of determination (R^2^) for both segments are calculated.
  - The α values are interpreted as DFA exponents separately for the short- and long-scale ranges.
(e) Recording and visualization of results.

### 3.4. Signal Reconstruction

Following the application of Detrended Fluctuation Analysis (DFA) to individual IMF components, the signals were reconstructed within two independent scaling ranges: local (α_1_) and global (α_2_). The objective of this step was to isolate signal components exhibiting significant and well-fitted temporal correlation patterns, thereby enabling their subsequent use in classification. To ensure that the reconstructed signals included only components with meaningful correlation properties, a conditional criterion based on two simultaneous conditions was implemented:

- α > 0.5—indicates the presence of positive temporal correlations (persistent signal), suggesting an ordered fluctuation structure.
- *R*^2^ > 0.9—denotes a high quality of the linear regression fit in the log–log DFA plot, confirming the reliability of the estimated scaling exponent α.

For each IMF component, both scaling ranges were considered separately:

- If a component satisfied the criteria for α_1_ and *R*^2^*_*α_1_, it was included in the local reconstruction associated with short-term correlations.
- If a component met the conditions for α_2_ and *R*^2^*_*α_2_, it was incorporated into the global reconstruction. reflecting long-term temporal correlations.

### 3.5. Classification Methods

Following the multi-stage analysis of VAG signals, which included their decomposition via the EEMD method and assessment of temporal correlations using DFA, the classification of the obtained data becomes a crucial step in the diagnostic process. The objective of classification is to automatically distinguish healthy individuals from patients exhibiting symptoms of chondromalacia or knee osteoarthritis. In the presented approach, two complementary classification pathways were employed: one based on the analysis of numerical time–frequency and fluctuation features using Support Vector Machines (SVMs), and the other utilizing deep learning on scalogram images through Convolutional Neural Networks (CNNs). The application of both methods allows for the evaluation of classification performance depending on the type of data representation and signal characteristics.

The Support Vector Machine (SVM) is a supervised machine learning method widely used for classification and regression tasks, particularly effective when dealing with complex data and a limited number of features. The core concept of SVM involves finding a hyperplane that maximally separates data points belonging to different classes while maintaining the largest possible margin between the boundary examples, known as support vectors. In the present study, the SVM variant with a radial basis function (RBF) kernel was employed, enabling the modeling of nonlinear decision boundaries and better capturing the complex relationships between VAG signal features in healthy individuals and patients with osteoarthritis.

Convolutional Neural Networks (CNNs) currently represent one of the most effective deep learning tools for the analysis of image and signal data. Their architecture is designed to autonomously extract hierarchical features from input datasets. CNNs are now widely applied across numerous fields [52,53,54,55,56,57]. In the context of vibroacoustic signal analysis, CNNs have been employed as classifiers trained on time–frequency representations of VAG signals obtained via the Continuous Wavelet Transform (CWT). As a result of this transformation, the one-dimensional time-domain signal is converted into a two-dimensional time–frequency image. This image preserves both temporal and frequency information of the signal. Such representations enable leveraging the full potential of CNNs for classifying pathologies in knee joints.

The CNN architecture consists of a composition of layers comprising several types:

- Convolutional layers: Responsible for extracting local features from the input data by applying convolutions with learned filters. In the context of CWT images, these filters can identify patterns characteristic of fluctuations, microvibrations, and signal irregularities associated with cartilage degeneration.
- Activation layers: The most commonly used activation function is ReLU (Rectified Linear Unit), which introduces nonlinearity to the model and mitigates the vanishing gradient problem.
- Pooling layers: Typically, max-pooling layers reduce data dimensionality and increase the network’s robustness to small spatial shifts in features within the image.
- Fully connected layers: Employed at the end of the network, these layers transform the extracted features into a final classification vector.

To classify vibroacoustic signals represented as time–frequency images (CWTs), a convolutional neural network (CNN) was implemented in MATLAB R2024b software using the Deep Learning Toolbox. The architecture and training procedure were adapted to the characteristics of medical data and the limited number of samples by employing data augmentation and stratified cross-validation to maintain class balance. The implemented CNN model comprised three convolutional blocks with an increasing number of filters, structured as follows: convolutional layer (3 × 3 kernel, padding set to ‘same’), batch normalization, ReLU activation function, and max-pooling layer (2 × 2). After the third block, a Dropout layer with a rate of 0.4 was added to mitigate overfitting. The final layer included a softmax classifier with binary output classes (HC, OA). Each examined signal, x_α1 and x_α2, was transformed using the continuous wavelet transform (CWT). The resulting amplitude matrix was normalized and converted into an RGB image using the jet colormap. Images were resized according to configuration-dependent pixel dimensions and saved as input data. Class labels were assigned based on the original file names.

The classifier’s performance was evaluated using five-fold stratified cross-validation (k = 5). Training data were augmented with random rotations (±10°), scaling (90–110%), horizontal and vertical translations (±5 pixels), and horizontal flipping. Training employed the Adam optimizer for 30 epochs, with a batch size of 32 and L2 regularization of 0.01. For each fold, classification metrics were computed: Accuracy, Sensitivity, Specificity, Precision, Recall, F1-score, and Area Under the Curve (AUC). Additionally, Receiver Operating Characteristic (ROC) curves and confusion matrices were generated and saved as graphical files for documentation purposes.

To enhance sensitivity and interpretability, binary classification was performed using probability thresholding for the “HC” class with a cutoff set at 0.7. Cases with probabilities below this threshold were assigned to the OA class by default. This approach accounts for asymmetric classification costs, which is particularly important in medical applications where misclassifying a diseased patient as healthy may have significant clinical consequences. Testing on both preprocessed and raw data was conducted using an identical classification module for consistency.

To address the relatively small sample size and reduce the risk of model overfitting, the CNN architecture was intentionally kept lightweight, limiting the number of trainable parameters by using only three convolutional blocks and a single fully connected layer. Regularization strategies, including dropout, batch normalization, L2 weight penalization, and early stopping based on the validation loss, were incorporated. The combination of five-fold stratified cross-validation and data augmentation ensured that every sample was used both for training and validation, improving generalization performance and preventing the model from learning subject-specific artifacts.

### 3.6. Assumptions Adopted for Classification and Classifier Comparison Metrics

The classification process in this study was based on two independent analytical pathways: (1) numerical feature vectors derived from the time–frequency and fluctuation (DFA) domains, and (2) image-based representations of signals obtained via Continuous Wavelet Transform (CWT), classified using Convolutional Neural Networks (CNNs). The goal of the classification was to distinguish between two subject groups: healthy controls (HC) and patients diagnosed with knee osteoarthritis (OA). The analysis was conducted separately for two biomechanical conditions: closed kinetic chain (CKC) and open kinetic chain (OKC). The input data consisted of signals recorded from three sensor locations positioned around the knee joint (K1–K3), acquired during ten complete flexion–extension cycles within the 0–90° range.

For the support vector machine (SVM) classifier, a set of features describing signal dynamics was computed for the ten intrinsic mode functions (IMFs) obtained via the ensemble empirical mode decomposition (EEMD) method. These features included the centroid frequency (CF), the mean instantaneous frequency (MIF), signal energy (E), and parameters derived from detrended fluctuation analysis (DFA): short-term scaling exponent (α_1_), long-term scaling exponent (α_2_), and the breakpoint location.

To reduce dimensionality and standardize the interpretation of frequency bands, the ten IMFs were grouped into three frequency ranges corresponding to distinct levels of mechanical activity:

- High-frequency band (IMF1–IMF3, f > 300 Hz)—associated with microvibrations and contact-related phenomena at the articular surfaces;
- Mid-frequency band (IMF4–IMF6, 150–300 Hz)—reflecting the interaction of musculoskeletal structures;
- Low-frequency band (IMF7–IMF10, f < 150 Hz)—capturing global movement dynamics and macromechanical oscillations.

For each of these bands, average values of CF, MIF, E, and the relative position of the DFA breakpoint were calculated, resulting in a total of 12 features (3 bands × 4 parameters) used in subsequent analysis.

To preliminarily assess feature relevance, a statistical analysis was conducted. The normality of feature distributions within the OA (osteoarthritis) and HC (healthy control) groups was evaluated using the Lilliefors test. Due to violations of normality assumptions in several cases, the nonparametric Mann–Whitney U test (*p* < 0.05) was applied for between-group comparisons. Features that did not show statistically significant differences were excluded from further analysis. This step ensured that only features with demonstrable discriminative value were retained before addressing redundancy among predictors.

Subsequent feature selection was performed using the Neighborhood Component Analysis (NCA) algorithm to estimate feature weights and their contribution to the classification process. Features with weights below 0.05 were excluded, as these values correspond to less than ~60% of the average predictor importance (average weight of 0.083 for 12 features). Such low weights typically fall within the estimation variance of the NCA algorithm and may reflect redundancy or noise, thereby reducing model stability.

Following this selection, a five-feature input set was retained, comprising the relative DFA breakpoint location (breakpoint ratio), energy in the low and mid-frequency bands, and mean instantaneous frequency (MIF) in the low and high-frequency bands.

For the deep learning pipeline, two types of input data were prepared: processed signals—specifically the DFA exponents α_1_ and α_2_ reconstructed from IMFs meeting the criteria α > 0.5 and R^2^ > 0.9—and raw signals (RAW) subjected to preliminary filtering. Both data types were transformed using the continuous wavelet transform (CWT) into scalograms of 128 × 128 and 256 × 256 pixel resolution. The convolutional neural network (CNN) architecture employed consisted of three convolutional blocks (each comprising Conv–BatchNorm–ReLU–MaxPooling), followed by a Dropout layer (rate = 0.4) and a softmax classification layer for two output classes (HC, OA). Training was conducted using the Adam optimizer for 30 epochs with a batch size of 32 and L2 regularization set to 0.01. To enhance model generalizability, data augmentation was applied, including rotations (±10°), scaling (90–110%), translations (±5 pixels), and horizontal flipping. Model evaluation was performed using five-fold cross-validation.

To compare the performance of the two approaches (SVM and CNN), a consistent set of evaluation metrics was used: accuracy, sensitivity (recall), specificity, precision, F1-score, and the area under the ROC curve (AUC). Results were reported as mean ± standard deviation across five validation folds. Additional evaluation was provided through ROC curves and confusion matrices for each experimental configuration. This approach ensured the comparability of results and enabled a comprehensive assessment of the effectiveness of both classifier types under conditions involving varying knee joint dynamics and loading scenarios.

## 4. Results

This chapter presents the results of vibroacoustic signal (VAG) analyses conducted in a cohort of patients with knee osteoarthritis (OA) and a control group (HC). The findings are organized in accordance with the adopted research methodology, encompassing both the evaluation of parameters derived from EEMD and DFA, as well as the classification performance achieved using two distinct approaches. Results obtained using a support vector machine (SVM) classifier and convolutional neural networks (CNNs) are presented. The first part of the chapter discusses the characteristics of selected signal features, categorized by frequency ranges and biomechanical recording conditions (CKC, OKC). This is followed by the presentation of diagnostically relevant feature selection outcomes and classification performance with respect to the applied algorithms. The analysis is complemented by ROC curves, confusion matrices, and a comparison of classification quality metrics, allowing for a comprehensive assessment of the proposed approach’s utility in detecting pathological changes in articular cartilage.

### 4.1. Classification Analysis of VAG Signals Using Time–Frequency and Fluctuation-Based Features

In the initial stage of the classification analysis, a set of numerical features was employed, derived from specific frequency bands of the VAG signal. This set included the breakpoint location in the DFA profile, the energy of selected IMF components, the mean instantaneous frequency (MIF), and the centroid frequency (CF). These parameters reflect both the dynamics of temporal fluctuations and the distribution of energy and frequency activity within the signal, enabling a quantitative characterization of differences between healthy controls (HCs) and patients with knee osteoarthritis (OA).

Classification was performed using a support vector machine (SVM) with a radial basis function (RBF) kernel. Model performance was evaluated through five-fold stratified cross-validation, yielding an average classification accuracy of 87% ± 3% under CKC conditions and 76% ± 10% under OKC conditions. Table 1 presents the mean classification results (±SD) for the open kinetic chain (OKC) and closed kinetic chain (CKC) conditions. Table 1 presents the mean classification results (±SD) for the open kinetic chain (OKC) and closed kinetic chain (CKC) conditions.

**Figure 9:**
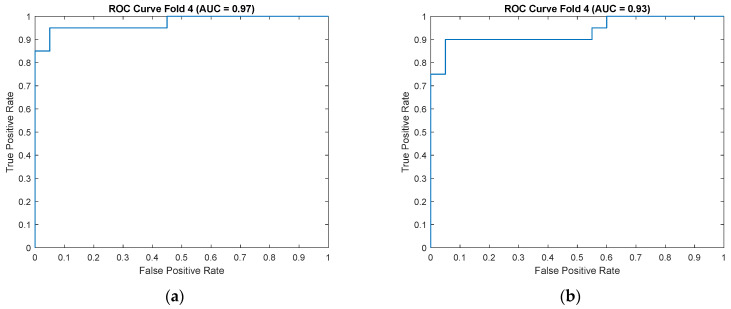
ROC curves under (**a**) closed kinetic chain (CKC) and (**b**) open kinetic chain (OKC) conditions.

**Figure 10:**
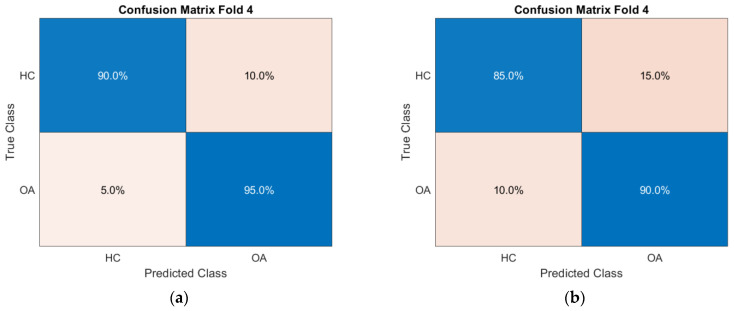
Confusion matrices under (**a**) closed kinetic chain (CKC) and (**b**) open kinetic chain (OKC) conditions.

For enhanced visualization of classifier performance, the subsequent sections also present the obtained ROC curves (Figure 9) and confusion matrices (Figure 10) for the CKC and OKC conditions. These results correspond to the fold with the highest classification accuracy achieved during the experiment. They indicate a distinctly better class separation under CKC conditions, further confirming the potential of this configuration for classification tasks based on vibroacoustic signals.

The applied feature set, comprising selected time–frequency parameters and DFA fluctuation indices, represents a novel approach in the analysis of VAG signals for classifying functional states of the knee joint. To date, these features have not been utilized as inputs for classifiers in this context, rendering them a promising direction for further research. Their combination not only enables high-accuracy differentiation between OA and HC groups but also may serve as the foundation for future tools to support the diagnosis of knee osteoarthritis.

### 4.2. Time–Frequency Representation of VAG Signals as Input to CNN in Classification Tasks

**Figure 11:**
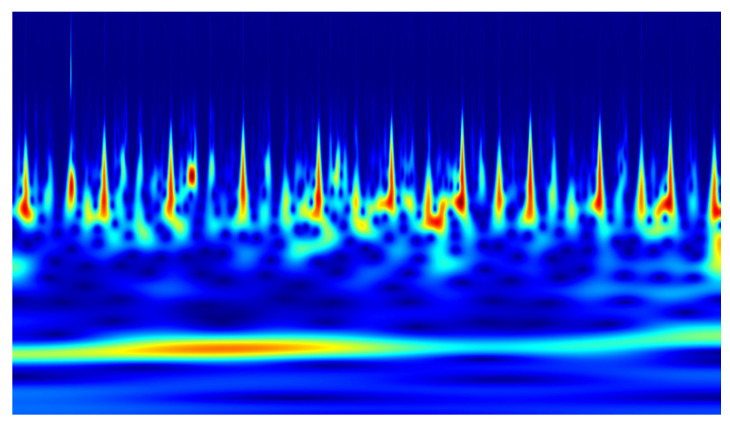
Scalogram α_1_, patellofemoral joint, CKC HC, post-processed data.

**Figure 12:**
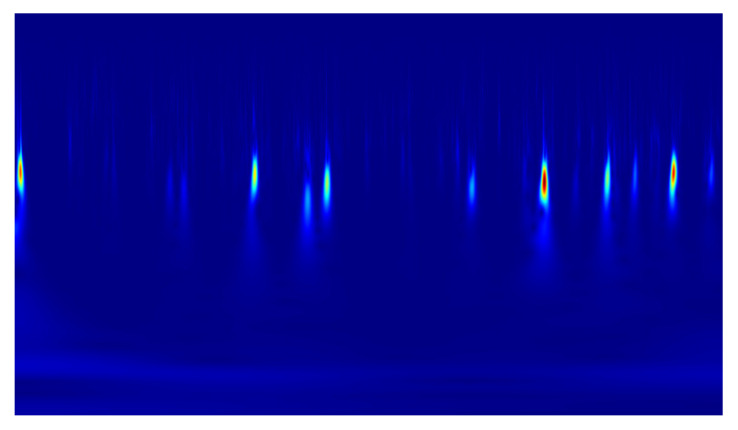
Scalogram α_1_, patellofemoral joint, CKC OA, post-processed data.

**Figure 13:**
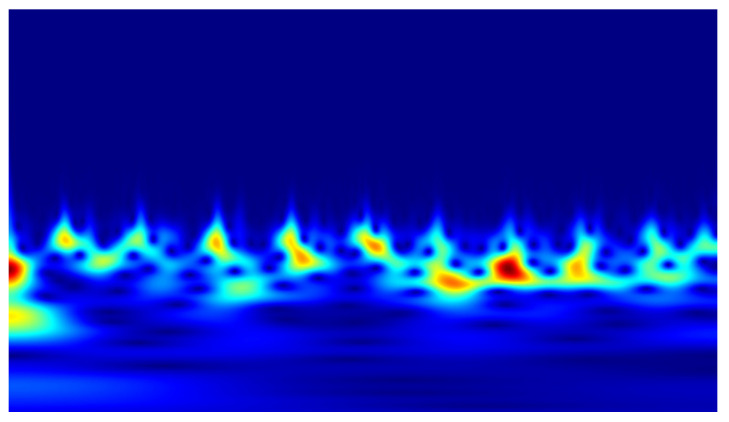
Scalogram α_2_, patellofemoral joint, CKC HC, post-processed data.

**Figure 14:**
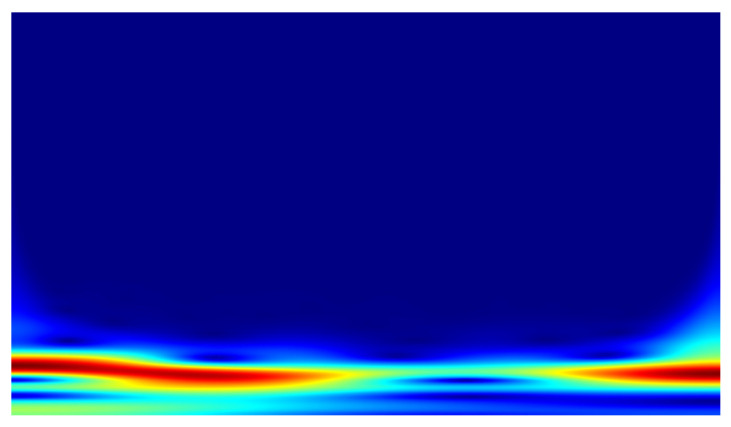
Scalogram α_2_, patellofemoral joint, CKC OA, post-processed data.

**Figure 15:**
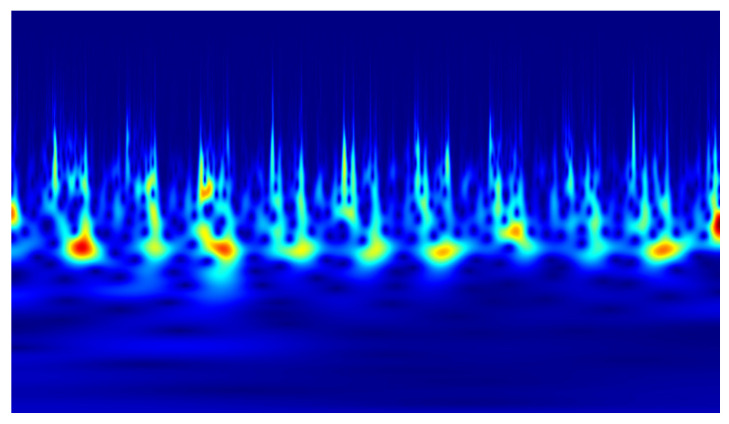
Scalogram, patellofemoral joint, CKC HC, raw data.

**Figure 16:**
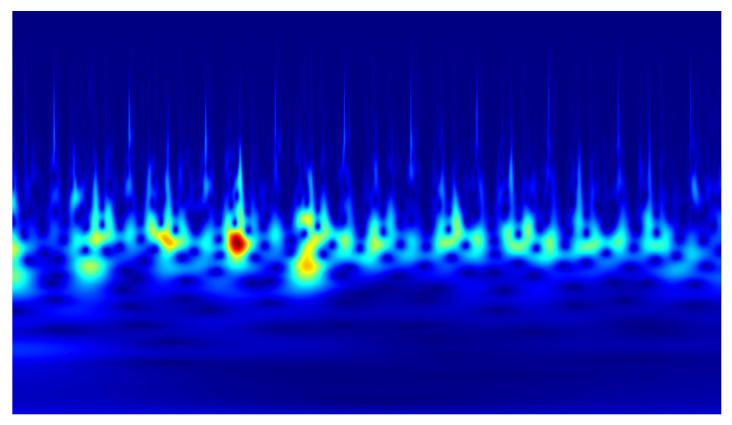
Scalogram, patellofemoral joint, CKC OA, raw data.

In the conducted study, classification was performed using convolutional neural networks (CNNs) applied to scalograms generated via continuous wavelet transform (CWT). Identical scale parameter values were used throughout the analysis. The experiments included both a group of signals exhibiting local correlations (α_1_) and signals reconstructed from components showing long-range correlations (α_2_). Example scalograms of the reconstructed signals (at a higher resolution than used in the classification experiments) are shown in Figure 11, Figure 12, Figure 13 and Figure 14. For comparison, scalograms of raw data are presented in Figure 15 and Figure 16. To enhance clarity in the publication, the visualizations were additionally rendered at increased resolution.

The generated scalograms were used as input for independent CNN classifiers. To balance classification accuracy and computational efficiency, experiments were conducted using low input image resolutions (128 × 128 and 256 × 256 pixels). This approach aimed to evaluate the classification capabilities in scenarios where image-based analysis must be performed on standard computers commonly available in medical laboratories, without requiring specialized computational hardware (e.g., CUDA-enabled GPUs). Although higher input resolutions may potentially improve performance, the primary objective was to assess whether the developed algorithm maintains sufficient efficiency and accuracy to enable diagnostic data analysis within time frames acceptable for clinical use. This consideration is particularly relevant in settings with limited hardware resources. Table 2 presents the execution times of the algorithm, tested on a computer with the specified hardware configuration.

As can be readily observed, the algorithm execution times involving signal processing are significantly longer, which is attributable to the time required to perform the necessary transformations.

Classification experiments using convolutional neural networks (CNNs) were conducted to evaluate the suitability of time–frequency representations of VAG signals for detecting knee osteoarthritis. The network inputs were prepared as the sum of the signals in channels K1, K2, and K3, in two variants:

1. Processed data (xα_1_ and xα_2_): VAG signals reconstructed from IMF components meeting correlation criteria (α_1_/α_2_ > 0.5, R^2^ > 0.9), subsequently subjected to wavelet transform and encoded as images.
2. Raw data: Raw signals (after low-pass filtering), also transformed using the CWT method.

For each configuration, data were analyzed under closed (CKC) and open (OKC) kinetic chain conditions, using different input image resolutions (128 × 128 and 256 × 256 pixels). Networks were trained using five-fold cross-validation (k = 5), and performance was evaluated based on the following metrics: accuracy, sensitivity, specificity, precision, F1-score, and AUC. The obtained results are summarized in Table 3.

Table 3 presents the averaged CNN classification results from five-fold cross-validation (k = 5) for various input data configurations. The analysis included signals reconstructed based on DFA parameters (α_1_ and α_2_), raw data (RAW), two time–frequency representation resolutions (128 × 128 and 256 × 256 pixels), and two biomechanical conditions (open OKC and closed CKC kinetic chains). The highest average classification performance was achieved for α_1_ data under OKC conditions at a resolution of 256 × 256 pixels, with Accuracy of 0.865 ± 0.030 and AUC of 0.899 ± 0.008. Very good metric values were also obtained for the CKC, α_1_, 256 × 256 px configuration (Accuracy: 0.840 ± 0.038, AUC: 0.891 ± 0.021), confirming the diagnostic value of local temporal fluctuations (α_1_). In contrast, α_2_ data, based on global temporal correlations, showed markedly lower classification metrics, particularly for the OKC/256 × 256 px configuration, where Accuracy was 0.706 ± 0.052 and AUC was 0.761 ± 0.066. Raw data (raw) exhibited the lowest stability and classification performance. The highest average Accuracy and F1-score values for raw data did not exceed approximately 0.68 and were accompanied by high variability (e.g., raw/OKC/256 px: Accuracy 0.544 ± 0.262). It is noteworthy that individual folds sometimes indicated record-high results, such as for OKC/α_1_/128 × 128 px with Accuracy = 0.927 and AUC = 0.914, and CKC/α_1_/256 × 256 px with Accuracy = 0.902 and AUC = 0.930. However, the analysis of averages across the entire validation suggests these were outliers likely due to random data distribution within specific folds, underscoring the importance of using averaged results as the basis for model evaluation.

**Figure 17:**
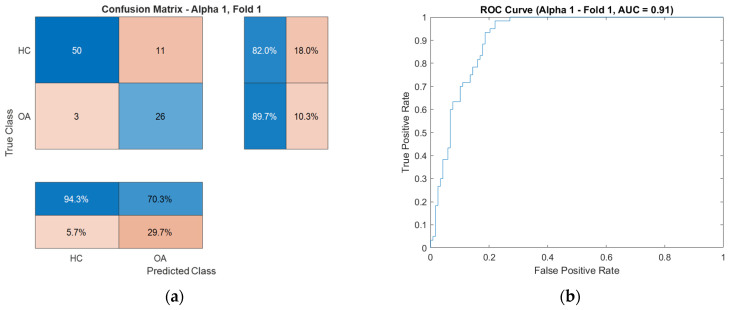
Configuration OKC α1, 128 × 128 px: (**a**) Confusion Matrix; (**b**) ROC Curve.

**Figure 18:**
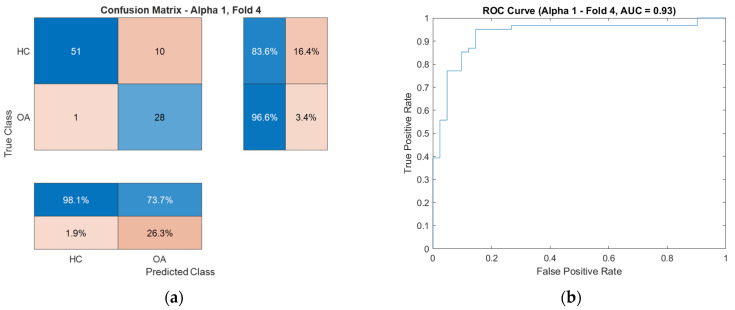
Configuration CKC α1, 256 × 256 px: (**a**) Confusion Matrix; (**b**) ROC Curve.

To illustrate the characteristics of these exceptional cases, confusion matrices and ROC curves for the OKC configuration are presented in Figure 17, respectively, while those for the CKC configuration are shown in Figure 18.

The use of higher resolution (256 × 256 px) time–frequency images resulted in a clear improvement in classification accuracy for processed data utilizing local DFA correlations (α_1_). In both biomechanical conditions (CKC and OKC), an increase in accuracy of approximately 6–7 percentage points was observed, accompanied by a reduction in standard deviation. For α_2_ reconstructions, the improvement was less pronounced and inconsistent, suggesting a lower sensitivity of global correlations to the level of detail in the representation.

In the case of raw data, increasing the input image resolution did not enhance classification results; instead, it led to greater variability in metrics and a decrease in effectiveness. This effect can be interpreted as a consequence of the lack of selection of diagnostically relevant components, whereby higher resolution exposes random fluctuations and noise. In a sense, this phenomenon resembles the inverse of filtering, as it disperses and obscures relevant information instead of isolating it from the signal.

As a result, the CNN classifier receives an excess of local, irregular patterns that carry no diagnostic value and may lead to erroneous generalizations. This observation highlights the importance of the preliminary step of selective signal decomposition and reconstruction prior to their transformation into time–frequency representations.

## 5. Discussion

Dysfunction of the knee joint in the course of osteoarthritis (OA) manifests not only in structural changes but also in impairments of biomechanics and neuromuscular control. This is clearly reflected in the recorded vibroacoustic signals, which represent the complex interplay of forces acting within the joint, the quality of the articular surfaces, and the coordination of muscular activity. Therefore, the analyzed signals can capture subtle changes resulting from the progressive degeneration of tissues as well as from the impairment of joint stabilizing mechanisms, making them a valuable source of information about the functional status of the musculoskeletal system.

Movements performed in closed and open kinematic chains (CKC vs. OKC) differ significantly in terms of load, stabilization, and activation of cooperating muscles. In the context of OA, coordination disturbances and irregular micromovements of the articular surfaces occur, leading to alterations in the characteristics of the generated vibration and acoustic signals. These changes manifest within specific frequency ranges, enabling their identification and interpretation through time–frequency analysis.

For this reason, the analysis of signal dynamics using methods such as EEMD and DFA is justified by the biomechanical nature of the problem. Features such as energy, centroidal frequency, and DFA parameters (α_1_, α_2_, breakpoint) can reflect disturbances in the cyclicity and regularity of joint movement, which are characteristic of osteoarthritis progression. Their variability may be associated with impaired stabilization, disruptions in motor control, and changes in the interaction of articular surfaces.

The results of the conducted SVM classification analysis based on features extracted from designated frequency bands showed that these differences are particularly pronounced under CKC conditions, where increased joint stabilization and compensatory muscle activations highlight mechanical disturbances more clearly than during free movement in OKC. The classification performed using the support vector machine (SVM) confirmed the superiority of CKC conditions over OKC in differentiating between OA and HC groups. For the CKC configuration, an average classification accuracy of 87% ± 3% was achieved, with sensitivity at 88% ± 8% and specificity at 86% ± 8%. These values are higher than those obtained for the OKC configuration, where accuracy averaged 76% ± 10%, and specificity dropped to 67% ± 16%, indicating greater difficulty in detecting OA patients under conditions of lower joint stabilization (OKC). Notably, the area under the curve (AUC) was also significantly higher for CKC (0.91 ± 0.04 vs. 0.85 ± 0.05), confirming the classifier’s better ability to distinguish classes across varying decision thresholds. This difference may be explained by increased compensatory activity under closed-chain conditions, which more clearly reveals the pathomechanical features of OA in the analyzed signals. Importantly, the precision and F1-score achieved in CKC also exceeded those in OKC, indicating greater stability and usefulness of the feature set extracted from this biomechanical configuration.

From a biomechanical perspective, disturbances associated with knee osteoarthritis (OA) also manifest in changes in the temporal scales of local and global correlations within the signal structure. These alterations may result from, among other factors, irregular joint surface contacts, stiffness, or compensatory muscle activation patterns. To isolate these phenomena, DFA was conducted separately for each intrinsic mode function (IMF) component, followed by the reconstruction of two signals representing dominant short-range and long-range correlations, respectively.

For both reconstructed signals (α_1_ and α_2_), under CKC and OKC conditions, scalograms were generated using continuous wavelet transform (CWT) at resolutions of 128 × 128 and 256 × 256 pixels. These prepared images served as input data for classification using convolutional neural networks (CNNs). The classification results showed that the best performance was achieved for the CKC configuration with scalograms based on the α_1_ signal at the higher resolution (256 × 256 px), yielding an accuracy of 84.0% ± 3.8% and an AUC of 0.891 ± 0.021. In comparison, the accuracy for the α_2_ signal under the same conditions dropped to 71.7% ± 3.6%. It is also noteworthy that a clear advantage of the α_1_ signal over α_2_ was observed for the OKC data, with classification accuracies of 86.5% ± 3.0% versus 70.6% ± 5.2% (for 256 × 256 px resolution). Processing raw signals (RAW), without reconstruction, resulted in significantly lower performance, below 64% for most configurations. This highlights the crucial role of preliminary signal processing using EEMD and DFA. Thanks to these methods, representations (α_1_ and α_2_) capturing local and global signal dynamics were obtained, which translated into a marked improvement in classification effectiveness compared to raw data.

Regarding the signal processing pipeline itself, both the EEMD and DFA methods require the selection of specific parameters (e.g., ensemble size, noise amplitude, window size and segmentation for DFA, or scaling ranges), which can influence the results obtained. In this study, these parameters were chosen based on prior pilot analyses and tested for stability; however, full optimization under the variable conditions of VAG signals requires further investigation.

The obtained results confirm that features extracted using EEMD and DFA methods can serve as valuable components in the non-invasive assessment of knee joint function. The application of such tools in VAG analysis opens promising avenues for future solutions supporting the diagnosis of osteoarthritis, particularly in clinical and rehabilitation settings. A comparative summary of recent state-of-the-art studies on acoustic and vibroarthrographic signal analysis applied to knee osteoarthritis detection is presented in Table 4. This overview highlights the methodological evolution from classical frequency-domain processing toward hybrid and deep learning approaches integrating multiscale decomposition and lightweight CNN architectures.

Recent studies have emphasized the importance of optimizing convolutional architectures toward lightweight, resource-efficient models capable of real-time inference in clinical environments. Modern frameworks such as MobileNetV3, EfficientNetV2, and ShuffleNetV2 have demonstrated comparable diagnostic accuracy to classical CNNs while reducing computational load by 40–70%, which is crucial for portable and point-of-care applications [65,66]. These models utilize depth-wise separable convolutions and neural architecture search (NAS) techniques, providing a favorable trade-off between inference time and accuracy without the need for GPU-based systems [60].

In the context of joint acoustic and vibroarthrographic (VAG) analysis, recent research (e.g., Yuan et al., 2025 [64]) confirmed that spectrogram-based lightweight CNNs can achieve high diagnostic performance while maintaining low latency, which makes them suitable for embedded and wearable diagnostic systems. Similarly, the SpectroNet and EEMD-DFA-CNN hybrid models proposed in [21,22] demonstrated that combining multiscale signal decomposition with compact deep learning architectures preserves accuracy (AUC > 0.9) while reducing processing time by over 50%.

Accordingly, future work will focus on benchmarking the proposed hybrid CNN against these lightweight deep architectures, incorporating pruning, quantization, and real-time CWT conversion to achieve near-instantaneous signal-to-diagnosis workflows. Such optimization will enable deployment in portable and bedside clinical systems, supporting on-site musculoskeletal assessments with minimal computational requirements.

## 6. Limitations and Future Plans

One of the main limitations of the conducted analysis is the necessity to balance the resolution of the generated scalograms with the time and computational resources required for their processing. Higher-resolution scalograms can more accurately represent the complex time–frequency structure of the signal and capture subtle diagnostic patterns; however, their effective analysis demands the use of specialized GPU units and parallel processing technologies such as CUDA. In practice, this may limit the scalability of the solution or its availability in clinical or research settings with limited hardware infrastructure.

Another important limitation concerns the relatively small size of the dataset, which is a common challenge in clinical vibroacoustic research. Although stratified cross-validation and extensive data augmentation were implemented, and the complexity of the machine learning models was intentionally constrained through dropout, batch normalization, L2 regularization, and a limited number of trainable parameters to prevent overfitting, the sample size may still affect the statistical power and generalizability of the obtained results. Therefore, the current study should be interpreted as a feasibility-oriented proof-of-concept, rather than a fully clinically validated diagnostic tool. Future studies will expand the patient cohort and include multi-center recruitment to improve classifier stability and population representativeness.

Although the proposed hybrid framework combining EEMD, DFA, and CNN-based analysis demonstrated high diagnostic accuracy, several aspects require further investigation. First, the present study focused primarily on binary classification (healthy vs. osteoarthritic joints), which does not fully reflect the continuum of cartilage degeneration observed in clinical practice. Future research should therefore aim to develop a multi-level classification system that corresponds to established clinical grading scales of cartilage damage, such as the Outerbridge, ICRS, or Kellgren–Lawrence systems. This approach will allow the algorithm to differentiate not only between healthy and pathological states, but also between early, moderate, and advanced stages of cartilage degradation.

To achieve reliable correlation between signal-based classifications and intraoperative or radiological grading, multi-modal data integration is planned—combining vibroacoustic analysis with MRI-based T2 or T1ρ mapping and arthroscopic evaluations. Enlarging the dataset and incorporating longitudinal recordings will also enable the assessment of disease progression and the sensitivity of VAG-derived parameters to subtle degenerative changes. Additionally, model transferability to portable and wearable diagnostic systems will be explored to support real-time, stage-aware clinical assessment.

## 7. Conclusions

In this study, a complex, multi-stage analytical pipeline was employed, combining the decomposition of VAG signals using Ensemble Empirical Mode Decomposition (EEMD), extraction of time–frequency and nonlinear features (DFA), feature selection, and classification. The application of the EEMD method enabled the decomposition of the VAG signal into a set of Intrinsic Mode Functions (IMFs), representing different frequency bands relevant to the vibrational characteristics of the knee joint. By grouping the IMFs into three bands (high, mid, low), it was possible to reduce dimensionality while preserving the physiological relevance of the features, which was leveraged in feature-based classification.

The introduction of Detrended Fluctuation Analysis (DFA) as a nonlinear analysis tool allowed the consideration of long-range correlations within the signal. This is particularly important given the biomechanical complexity of the joint and its pathological alterations in osteoarthritis (OA). The variability in the values of α_1_ and α_2_, as well as the location of the breakpoint between these ranges, indicates distinct fluctuation structures in the OA and healthy control (HC) groups, confirmed by statistically significant differences.

The SVM classifier based on selected EEMD–DFA features demonstrated robust performance, particularly under CKC conditions, achieving an average accuracy of 87% with a high AUC value of 0.91. These results confirm that carefully chosen nonlinear and time–frequency parameters can provide reliable discrimination between OA and HC groups, highlighting their potential as interpretable biomarkers for sensor-based cartilage damage detection.

In parallel, classification using Convolutional Neural Networks (CNNs) on the scalograms of IMFs was conducted as a complementary approach. These scalograms retain both time and frequency information, enabling CNNs to autonomously extract patterns characteristic of pathological vibrations. Higher classification performance observed under closed kinetic chain (CKC) conditions, for both feature-based classifiers and CNNs, suggests that CKC conditions better reveal pathological changes in joint vibrations.

The joint analysis of feature-based classifiers and CNN-based models indicates that, although these methods operate on different data representations, both are capable of identifying significant differences between the groups. The integration of EEMD, DFA, and CNN highlights the potential of a multi-stage approach to biomechanical signal analysis, combining the interpretability and transparency of handcrafted features with the classification power of deep learning.

## Figures and Tables

**Table 1:** Mean classification results (±SD) for the open and closed kinetic chain conditions.

Configuration	Accuracy	Sensitivity	Specificity	Precision	Recall	F1-Score
CKC (K1+K2+K3)	0.87 ± 0.03	0.88 ± 0.08	0.86 ± 0.08	0.87 ± 0.05	0.88 ± 0.08	0.87 ± 0.03
OKC (K1+K2+K3)	0.76 ± 0.10	0.84 ± 0.06	0.67 ± 0.16	0.74 ± 0.10	0.84 ± 0.06	0.78 ± 0.08

**Table 2:** Recorded algorithm execution times.

Configuration	Resolution	Signal Type	Execution Time [s]
CKC, α1 + α2	128 × 128	post-processed data	1438.989
OKC, α1 + α2	128 × 128	post-processed data	1742.950
CKC, α1 + α2	256 × 256	post-processed data	3384.791
OKC, α1 + α2	256 × 256	post-processed data	3655.883
CKC, α1 + α2	128 × 128	raw data	571.461
OKC, α1 + α2	128 × 128	raw data	452.108
CKC, α1 + α2	256 × 256	raw data	1510.297
OKC, α1 + α2	256 × 256	raw data	1532.978

**Table 3:** Mean classification results (±SD) of CNN for α_1_, α_2_, and raw data (CKC, OKC; 128/256 px).

Configuration	Accuracy	Sensitivity	Specificity	Precision	Recall	F1-Score
CKC, α1, 128 × 128 px	0.779 ± 0.064	0.844 ± 0.107	0.801 ± 0.052	0.798 ± 0.057	0.844 ± 0.107	0.799 ± 0.033
CKC, α2, 128 × 128 px	0.685 ± 0.053	0.674 ± 0.045	0.679 ± 0.068	0.690 ± 0.083	0.714 ± 0.136	0.687 ± 0.046
CKC, α1, 256 × 256 px	0.840 ± 0.038	0.855 ± 0.052	0.846 ± 0.030	0.873 ± 0.023	0.855 ± 0.052	0.861 ± 0.039
CKC, α2, 256 × 256 px	0.717 ± 0.036	0.728 ± 0.072	0.813 ± 0.089	0.708 ± 0.033	0.764 ± 0.053	0.721 ± 0.038
OKC, α1, 128 × 128 px	0.800 ± 0.070	0.801 ± 0.056	0.908 ± 0.063	0.733 ± 0.031	0.801 ± 0.056	0.804 ± 0.068
OKC, α2, 128 × 128 px	0.724 ± 0.036	0.740 ± 0.037	0.868 ± 0.081	0.741 ± 0.052	0.758 ± 0.011	0.776 ± 0.027
OKC, α1, 256 × 256 px	0.865 ± 0.030	0.868 ± 0.010	0.889 ± 0.036	0.849 ± 0.022	0.867 ± 0.009	0.839 ± 0.024
OKC, α2, 256 × 256 px	0.706 ± 0.052	0.831 ± 0.107	0.814 ± 0.095	0.727 ± 0.031	0.799 ± 0.140	0.759 ± 0.097
Raw, CKC, 128 × 128 px	0.629 ± 0.087	0.603 ± 0.204	0.588 ± 0.197	0.715 ± 0.040	0.603 ± 0.204	0.656 ± 0.142
Raw, CKC, 256 × 256 px	0.602 ± 0.046	0.621 ± 0.186	0.568 ± 0.206	0.708 ± 0.056	0.621 ± 0.186	0.639 ± 0.095
Raw, OKC, 128 × 128 px	0.676 ± 0.029	0.884 ± 0.074	0.310 ± 0.172	0.697 ± 0.045	0.884 ± 0.074	0.776 ± 0.018
Raw, OKC, 256 × 256 px	0.544 ± 0.262	0.556 ± 0.219	0.524 ± 0.035	0.643 ± 0.262	0.556 ± 0.160	0.564 ± 0.087

**Table 4:** Summary of recent state-of-the-art (SOTA) approaches for acoustic and vibroarthrographic (VAG/AE) signal analysis in knee osteoarthritis (OA) diagnostics.

Year	Authors [DOI]	Signal Analysis Method	Classification/Dimensionality Reduction Technique	Main Findings and Conclusions
2019	Whittingslow D.C. et al. [58]	Acoustic emissions (AEs) + kinematic XROMM analysis in a cadaveric knee model	b-value analysis + joint geometry correlation	Demonstrated that minimal inter-articular distance strongly correlates with AE amplitude increases—laying the groundwork for AE as a biomarker of joint structural health.
2018	Goossens P. et al. [59]	Knee Acoustic Emissions (KAEs) in Juvenile Idiopathic Arthritis (JIA)	Gradient-boosting (soft classifier)	Developed a ‘Knee Audio Score’ distinguishing JIA from controls; validation in an extended cohort (n = 116) achieved 87.7% testing accuracy.
2021	Olorunlambe K.A. et al. [60]	Acoustic emission (AE) in tribological and orthopedic diagnostics	PCA + supervised ML (k-NN, BP NN)	Classified adhesive vs. abrasive wear with 98% accuracy; confirmed AE potential for biomechanical and implant diagnostics.
2023	Khan T.I. et al. [61]	AE from knee joints—statistical analysis + PCA	Gaussian Mixture Model (GMM) clustering	Compact AE clustering across four age groups; effective discrimination of OA from healthy subjects, enabling early detection.
2024	Richardson K.L., Nichols C.J. et al. [62]	Joint Acoustic Emissions (JAEs)—cross-validation across setups and populations	Supervised learning; cross-setup generalization	Validated JAEs as a generalizable predictor of joint health (AUC = 0.85–0.99); demonstrated effects of sensor placement and recording configuration on model transferability.
2024	Machrowska A. et al. [21]	Multiscale analysis: EEMD + DFA	CNN-based classification of chondromalacia	The EEMD-DFA approach enabled accurate classification of cartilage degeneration stages (AUC = 0.91); robust to small datasets.
2025	Kumar V. et al. [63]	Vibroarthrography (VAG): Hilbert–Huang + Wavelet analysis	Self-Organizing Maps (SOMs) + K-Means clustering	High cluster separation (Silhouette = 0.8, DBI = 0.33); correlation between signal complexity and OA progression.
2025	Machrowska A., Karpiński R. et al. [21]	Hybrid EEMD–DFA–CWT model	CNN + SVM (dual-path hybrid)	Integrating nonlinear analysis with CNNs improved cartilage damage detection sensitivity (AUC > 0.9); suitable for early OA screening.
2025	Yuan J., Zhang J. and Qian G. [64]	Spectrogram-based learning from Joint Acoustic Emissions (JAEs)	Deep CNN (SpectroNet-like architecture)	Proposed a 2D spectrogram-based deep learning framework for knee joint analysis; achieved high accuracy in classifying inflammatory and degenerative knee conditions.

## Data Availability

Data presented in this study are available from the corresponding authors upon request.
